# Supplementary material for: Modelling Skylarks (Alauda arvensis) to Predict Impacts of Changes in Land Management and Policy: Development and Testing of an Agent-Based Model
Source: PLoS One. 2013 Jun 6;8(6):e65803. doi: 10.1371/journal.pone.0065803 (PMC3675089; doi:10.1371/journal.pone.0065803)
Supplement: Supporting Information S4 — The skylark ODdox as a zipped archive. (ZIP) [file pone.0065803.s004.zip › Skylark_ODdox/class_position_map.html]

ALMaSS Skylark ODdox: PositionMap Class Reference


ALMaSS Skylark ODdox
 2.0


- Main Page
- Related Pages
- Classes
- Files

- Class List
- Class Index
- Class Hierarchy
- Class Members

Public Member Functions |
Public Attributes |
Protected Member Functions |
Protected Attributes

PositionMap Class Reference

Used to map locations of individuals for density estimates.
More...

`#include <positionmap.h>`

List of all members.

|  |  |
| --- | --- |
| Public Member Functions | |
| void | ClearMapValue (unsigned x, unsigned y) |
| int | GetMapDensity (unsigned x, unsigned y) |
| int | GetMapDensity (unsigned x, unsigned y, unsigned range) |
| bool | GetMapDensity32 (unsigned x, unsigned y) |
| int | GetMapDensity5x5 (unsigned x, unsigned y) |
| bool | GetMapPositive (unsigned x, unsigned y, unsigned range) |
| bool | GetMapPositiveB (unsigned x, unsigned y, unsigned range) |
| int | GetMapValue (unsigned x, unsigned y) |
| int | GetTotalN (int x, int y) |
| int | GetTotalSpace (int x, int y) |
|  | PositionMap (unsigned a\_size) |
|  | PositionMap (Landscape \*L) |
| void | SetMapValue (unsigned x, unsigned y) |
|  | ~PositionMap () |

|  |  |
| --- | --- |
| Public Attributes | |
| PointerInt | m\_maxx |
| PointerInt | m\_maxy |
| PointerInt | m\_TheBitMaskArray [65] |
| PointerInt | m\_TheBitMaskArray2 [65] |
| PointerInt \* | m\_TheMap |
| PointerInt | m\_xmaxx |

|  |  |
| --- | --- |
| Protected Member Functions | |
| PointerInt | BitCount (PointerInt n) |
| void | Init () |

|  |  |
| --- | --- |
| Protected Attributes | |
| Landscape \* | m\_ALandscape |

---

## Detailed Description

Used to map locations of individuals for density estimates.

---

## Constructor & Destructor Documentation

|  |  |  |  |  |  |
| --- | --- | --- | --- | --- | --- |
| PositionMap::PositionMap | ( | unsigned | *a\_size* | ) |  |

References \_\_3264minus0, \_\_3264minus1, Init(), m\_maxx, m\_maxy, m\_TheMap, and m\_xmaxx.

{

// must make sure that we have whole words

m\_xmaxx=a\_size;

m\_maxx=(m\_xmaxx+\_\_3264minus1)/\_\_3264minus0;

m\_maxy=m\_maxx;

m\_TheMap = new PointerInt[ m\_maxx\*m\_maxy ];

Init();

}

|  |  |  |  |  |  |
| --- | --- | --- | --- | --- | --- |
| PositionMap::PositionMap | ( | Landscape \* | *L* | ) |  |

References \_\_3264minus0, \_\_3264minus1, Init(), m\_ALandscape, m\_maxx, m\_maxy, m\_TheMap, m\_xmaxx, Landscape::SupplySimAreaHeight(), and Landscape::SupplySimAreaWidth().

{

m\_ALandscape = L;

// must make sure that we have whole words

m\_xmaxx=m\_ALandscape->SupplySimAreaWidth();

m\_maxx=(m\_xmaxx+\_\_3264minus1)/\_\_3264minus0;

m\_maxy=m\_ALandscape->SupplySimAreaHeight();

m\_TheMap = new PointerInt[ m\_maxx\*m\_maxy ];

Init();

}

|  |  |  |  |  |
| --- | --- | --- | --- | --- |
| PositionMap::~PositionMap | ( |  | ) |  |

References m\_TheMap.

{

delete m\_TheMap;

}

---

## Member Function Documentation

|  |  |  |  |  |  |
| --- | --- | --- | --- | --- | --- |
| PointerInt PositionMap::BitCount | ( | PointerInt | *n* | ) |  |

inlineprotected

Referenced by GetMapDensity().

{

unsigned int nCount=0 ;

for(; n; n&=(n-1)){

nCount++;

}

return nCount ;

}

|  |  |  |  |
| --- | --- | --- | --- |
| void PositionMap::ClearMapValue | ( | unsigned | *x*, |
|  |  | unsigned | *y* |
|  | ) |  |  |

References \_\_3264divide, \_\_3264minus1, m\_maxx, and m\_TheMap.

Referenced by Init().

{

PointerInt theBit= x & \_\_3264minus1;

// divide by 32

// Calculate index:

PointerInt index= (x>> \_\_3264divide )+(m\_maxx\*y);

m\_TheMap[index] &= ~(0x01 << theBit);

}

|  |  |  |  |
| --- | --- | --- | --- |
| int PositionMap::GetMapDensity | ( | unsigned | *x*, |
|  |  | unsigned | *y* |
|  | ) |  |  |

References \_\_3264divide, \_\_3264minus1, m\_maxx, and m\_TheMap.

{

if (y==0) y=1;

PointerInt TheTotal=0;

PointerInt theBit= (x-1) & \_\_3264minus1; // Identifies which bit to check

PointerInt index= (x>> \_\_3264divide )+(m\_maxx\*(y+1));

TheTotal+=((m\_TheMap[index] >> theBit) & 0x01);

index= (x>> \_\_3264divide )+(m\_maxx\*(y-1));

TheTotal+=((m\_TheMap[index] >> theBit) & 0x01);

index= (x>> \_\_3264divide )+(m\_maxx\*(y));

TheTotal+=((m\_TheMap[index] >> theBit) & 0x01);

theBit= (x+1) & \_\_3264minus1;

index= (x>> \_\_3264divide )+(m\_maxx\*(y+1));

TheTotal+=((m\_TheMap[index] >> theBit) & 0x01);

index= (x>> \_\_3264divide )+(m\_maxx\*(y-1));

TheTotal+=((m\_TheMap[index] >> theBit) & 0x01);

index= (x>> \_\_3264divide )+(m\_maxx\*(y));

TheTotal+=((m\_TheMap[index] >> theBit) & 0x01);

theBit= x & \_\_3264minus1;

index= (x>> \_\_3264divide )+(m\_maxx\*(y+1));

TheTotal+=((m\_TheMap[index] >> theBit) & 0x01);

index= (x>> \_\_3264divide )+(m\_maxx\*(y-1));

TheTotal+=((m\_TheMap[index] >> theBit) & 0x01);

return int(TheTotal);

}

|  |  |  |  |
| --- | --- | --- | --- |
| int PositionMap::GetMapDensity | ( | unsigned | *x*, |
|  |  | unsigned | *y*, |
|  |  | unsigned | *range* |
|  | ) |  |  |

References \_\_3264divide, \_\_3264minus0, \_\_3264minus1, BitCount(), m\_maxx, m\_maxy, m\_TheBitMaskArray, m\_TheBitMaskArray2, and m\_TheMap.

{

// THIS METHOD IS UNTESTED AND PROBABLY IS BUGGY!!

// This method is called with the topleft coord as x,y - will probably need range checking in the calling function!

// for negative co-ords

// Efficiency is high for large areas, for small areas then it is less efficient than testing each square

// NB this method will automatically wrap-round if it meets the edge of X co-ord or Y-Coord

PointerInt TheTotal=0;

PointerInt theBit= x & \_\_3264minus1; // Identifies which bit to check first e.g. 49 & 31 = 17

PointerInt index= ( x>> \_\_3264divide )+( m\_maxx \* ( y ) ); // This is the start index

PointerInt yextent=y+range;

if (yextent>m\_maxy) {

yextent=yextent-m\_maxy;

// This code is duplicated just to avoid the extra call and parameter pass

// NB BitCount is inline.

for (PointerInt yy=y; yy<yextent; yy++) { // Do all this for each y

PointerInt remain=range-(\_\_3264minus0-theBit); // e.g 55-17=38

if (remain>=0) { // Need to continue

// We need the rest of the 32 bits

TheTotal+=BitCount(m\_TheMap[index]& m\_TheBitMaskArray[theBit]); // NB if we are at the last bit then the mask is 0x08000 not 0x00001 !!

}

else {

// Stop this y here because remain is negative

// We need all the rest of the bits from our start point (theBit) to theBit+range

// e.g. theBit=17, remain = 3, we needs bits 17,18,19

TheTotal+=BitCount(m\_TheMap[index]& (m\_TheBitMaskArray[theBit]^m\_TheBitMaskArray[theBit+range]));

remain=0;

}

// Now if remain is >32 we need to take a whole PointerInt else only the low remain bits

while (remain>0) {

if (remain>\_\_3264minus0) {

TheTotal+=BitCount(m\_TheMap[++index]);

} else {

TheTotal+=BitCount(m\_TheMap[++index]& m\_TheBitMaskArray2[remain]); // Use a second array to avoid the extra operation of inverting the first

}

remain-=\_\_3264minus0;

}

}

yextent=m\_maxy;

}

for (PointerInt yy=y; yy<yextent; yy++) { // Do all this for each y

PointerInt remain=range-(\_\_3264minus0-theBit); // e.g 55-17=38

if (remain>=0) { // Need to continue

// We need the rest of the 32 bits

TheTotal+=BitCount(m\_TheMap[index]& m\_TheBitMaskArray[theBit]); // NB if we are at the last bit then the mask is 0x08000 not 0x00001 !!

}

else {

// Stop this y here because remain is negative

// We need all the rest of the bits from our start point (theBit) to theBit+range

// e.g. theBit=17, remain = 3, we needs bits 17,18,19

TheTotal+=BitCount(m\_TheMap[index]& (m\_TheBitMaskArray[theBit]^m\_TheBitMaskArray[theBit+range]));

remain=0;

}

// Now if remain is >32 we need to take a whole PointerInt else only the low remain bits

while (remain>0) {

if (remain>\_\_3264minus0) {

TheTotal+=BitCount(m\_TheMap[++index]);

} else {

TheTotal+=BitCount(m\_TheMap[++index]& m\_TheBitMaskArray2[remain]); // Use a second array to avoid the extra operation of inverting the first

}

remain-=\_\_3264minus0;

}

}

return int(TheTotal);

}

|  |  |  |  |
| --- | --- | --- | --- |
| bool PositionMap::GetMapDensity32 | ( | unsigned | *x*, |
|  |  | unsigned | *y* |
|  | ) |  |  |

References \_\_3264divide, \_\_3264minus1, m\_maxx, and m\_TheMap.

{

// Returns true if there is another animal in 32x32m

// IMPORTANT - this function does not include bounds checking

// Bounds checking on Y is needed before the call here

// this is an odd density method. It assumes that all that is important is

// whether there is another beetle within the 32m represented by each point

// So two beetles could be side by side, but in different uints and not kill

// each other, but conversely, 31 m apart, in the same one and kill each other

// This function is therefore only used as a speed optimisation

//

PointerInt index;

PointerInt theBit; // Identifies which bit to check

for (unsigned int yoff=y-16; yoff<y; yoff++)

{

index = (x>> \_\_3264divide )+(m\_maxx\*(yoff));

if (m\_TheMap[index]>0) return true;

}

// Special case for 'y'

theBit= x & \_\_3264minus1; // Identifies which bit to check

theBit=PointerInt(1)<<theBit;

index = (x>> \_\_3264divide )+(m\_maxx\*(y));;

if((m\_TheMap[index]^theBit)>0)

return true; // use XOR to remove the calling beetle

for (unsigned int yoff=y+1; yoff<y+16; yoff++)

{

index = (x>> \_\_3264divide )+(m\_maxx\*(yoff));

if (m\_TheMap[index]>0) return true;

}

return false;

}

|  |  |  |  |
| --- | --- | --- | --- |
| int PositionMap::GetMapDensity5x5 | ( | unsigned | *x*, |
|  |  | unsigned | *y* |
|  | ) |  |  |

References \_\_3264divide, \_\_3264minus1, m\_maxx, and m\_TheMap.

{

if (y<2) y=2; // Don't run over the world backwards - big -ve number results

if (x<2) x=2;

PointerInt index;

PointerInt TheTotal=0;

PointerInt theBit= (x-2) & \_\_3264minus1; // Identifies which bit to check

index = (x>> \_\_3264divide )+(m\_maxx\*(y+2));

TheTotal+=((m\_TheMap[index] >> theBit) & 0x01);

index= (x>> \_\_3264divide )+(m\_maxx\*(y+1));

TheTotal+=((m\_TheMap[index] >> theBit) & 0x01);

index= (x>> \_\_3264divide )+(m\_maxx\*(y));

TheTotal+=((m\_TheMap[index] >> theBit) & 0x01);

index= (x>> \_\_3264divide )+(m\_maxx\*(y-1));

TheTotal+=((m\_TheMap[index] >> theBit) & 0x01);

index= (x>> \_\_3264divide )+(m\_maxx\*(y-2));

TheTotal+=((m\_TheMap[index] >> theBit) & 0x01);

theBit= (x-1) & \_\_3264minus1; // Identifies which bit to check

index = (x>> \_\_3264divide )+(m\_maxx\*(y+2));

TheTotal+=((m\_TheMap[index] >> theBit) & 0x01);

index= (x>> \_\_3264divide )+(m\_maxx\*(y+1));

TheTotal+=((m\_TheMap[index] >> theBit) & 0x01);

index= (x>> \_\_3264divide )+(m\_maxx\*(y));

TheTotal+=((m\_TheMap[index] >> theBit) & 0x01);

index= (x>> \_\_3264divide )+(m\_maxx\*(y-1));

TheTotal+=((m\_TheMap[index] >> theBit) & 0x01);

index= (x>> \_\_3264divide )+(m\_maxx\*(y-2));

TheTotal+=((m\_TheMap[index] >> theBit) & 0x01);

theBit= x & \_\_3264minus1;

index = (x>> \_\_3264divide )+(m\_maxx\*(y+2));

TheTotal+=((m\_TheMap[index] >> theBit) & 0x01);

index= (x>> \_\_3264divide )+(m\_maxx\*(y+1));

TheTotal+=((m\_TheMap[index] >> theBit) & 0x01);

index= (x>> \_\_3264divide )+(m\_maxx\*(y-1));

TheTotal+=((m\_TheMap[index] >> theBit) & 0x01);

index= (x>> \_\_3264divide )+(m\_maxx\*(y-2));

TheTotal+=((m\_TheMap[index] >> theBit) & 0x01);

theBit= (x+1) & \_\_3264minus1;

index = (x>> \_\_3264divide )+(m\_maxx\*(y+2));

TheTotal+=((m\_TheMap[index] >> theBit) & 0x01);

index= (x>> \_\_3264divide )+(m\_maxx\*(y+1));

TheTotal+=((m\_TheMap[index] >> theBit) & 0x01);

index= (x>> \_\_3264divide )+(m\_maxx\*(y));

TheTotal+=((m\_TheMap[index] >> theBit) & 0x01);

index= (x>> \_\_3264divide )+(m\_maxx\*(y-1));

TheTotal+=((m\_TheMap[index] >> theBit) & 0x01);

index= (x>> \_\_3264divide )+(m\_maxx\*(y-2));

TheTotal+=((m\_TheMap[index] >> theBit) & 0x01);

theBit= (x+2) & \_\_3264minus1;

index = (x>> \_\_3264divide )+(m\_maxx\*(y+2));

TheTotal+=((m\_TheMap[index] >> theBit) & 0x01);

index= (x>> \_\_3264divide )+(m\_maxx\*(y+1));

TheTotal+=((m\_TheMap[index] >> theBit) & 0x01);

index= (x>> \_\_3264divide )+(m\_maxx\*(y));

TheTotal+=((m\_TheMap[index] >> theBit) & 0x01);

index= (x>> \_\_3264divide )+(m\_maxx\*(y-1));

TheTotal+=((m\_TheMap[index] >> theBit) & 0x01);

index= (x>> \_\_3264divide )+(m\_maxx\*(y-2));

TheTotal+=((m\_TheMap[index] >> theBit) & 0x01);

return int(TheTotal);

}

|  |  |  |  |
| --- | --- | --- | --- |
| bool PositionMap::GetMapPositive | ( | unsigned | *x*, |
|  |  | unsigned | *y*, |
|  |  | unsigned | *range* |
|  | ) |  |  |

References \_\_3264divide, \_\_3264minus0, \_\_3264minus1, m\_maxx, m\_maxy, m\_TheBitMaskArray, m\_TheBitMaskArray2, and m\_TheMap.

{

// THIS METHOD IS UNTESTED AND PROBABLY IS BUGGY!!

// This method is called with the topleft coord as x,y - will probably need range checking in the calling function!

// for negative co-ords

// Efficiency is high for large areas, for small areas then it is less efficient than testing each square

// NB this method will automatically wrap-round if it meets the edge of X co-ord or Y-Coord

PointerInt theBit= PointerInt(x) & \_\_3264minus1; // Identifies which bit to check first e.g. 49 & 31 = 17

PointerInt index= ( PointerInt(x)>> \_\_3264divide )+( m\_maxx \* ( PointerInt(y) ) ); // This is the start index

PointerInt yextent=y+range;

if (yextent>m\_maxy) {

yextent=yextent-m\_maxy;

// This code is duplicated just to avoid the extra call and parameter pass

// NB BitCount is inline.

for (unsigned yy=y; yy<yextent; yy++) { // Do all this for each y

PointerInt remain=range-(\_\_3264minus0-theBit); // e.g 55-17=38

if (remain>=0) { // Need to continue

// We need the rest of the 32 bits

if ((m\_TheMap[index]& m\_TheBitMaskArray[theBit])!=0) return true; // NB if we are at the last bit then the mask is 0x08000 not 0x00001 !!

}

else {

// Stop this y here because remain is negative

// We need all the rest of the bits from our start point (theBit) to theBit+range

// e.g. theBit=17, remain = 3, we needs bits 17,18,19

if ((m\_TheMap[index]& (m\_TheBitMaskArray[theBit]^m\_TheBitMaskArray[theBit+range]))!=0) return true;

remain=0;

}

// Now if remain is >32 we need to take a whole PointerInt else only the low remain bits

while (remain>0) {

if (remain>\_\_3264minus0) {

if(m\_TheMap[++index]!=0) return true;

} else {

if ((m\_TheMap[++index]& m\_TheBitMaskArray2[remain])!=0) return true; // Use a second array to avoid the extra operation of inverting the first

}

remain-=\_\_3264minus0;

}

}

yextent=m\_maxy;

}

for (PointerInt yy=y; yy<yextent; yy++) { // Do all this for each y

PointerInt remain=range-(\_\_3264minus0-theBit); // e.g 55-17=38

if (remain>=0) { // Need to continue

// We need the rest of the 32 bits

if ((m\_TheMap[index]& m\_TheBitMaskArray[theBit])!=0) return true; // NB if we are at the last bit then the mask is 0x08000 not 0x00001 !!

}

else {

// Stop this y here because remain is negative

// We need all the rest of the bits from our start point (theBit) to theBit+range

// e.g. theBit=17, remain = 3, we needs bits 17,18,19

if ((m\_TheMap[index]& (m\_TheBitMaskArray[theBit]^m\_TheBitMaskArray[theBit+range]))!=0) return true;

remain=0;

}

// Now if remain is >32 we need to take a whole PointerInt else only the low remain bits

while (remain>0) {

if (remain>\_\_3264minus0) {

if(m\_TheMap[++index]!=0) return true;

} else {

if ((m\_TheMap[++index]& m\_TheBitMaskArray2[remain])!=0) return true; // Use a second array to avoid the extra operation of inverting the first

}

remain-=\_\_3264minus0;

}

}

return false;

}

|  |  |  |  |
| --- | --- | --- | --- |
| bool PositionMap::GetMapPositiveB | ( | unsigned | *x*, |
|  |  | unsigned | *y*, |
|  |  | unsigned | *range* |
|  | ) |  |  |

References \_\_3264divide, \_\_3264minus1, m\_maxx, m\_maxy, m\_TheBitMaskArray, and m\_TheMap.

{

// This is a speed optimisation. We assume that we never need to go outside our actual uint

// so if our x is at 25 and range is 10 then we use x-3 (35-32) as the start point.

PointerInt theBit= x & \_\_3264minus1; // Identifies which bit to check first e.g. 49 & 31 = 17

PointerInt index= ( x>> \_\_3264divide )+( m\_maxx \* ( y ) ); // This is the start index

// Create the mask

//theBit has 0-31

PointerInt n1=theBit+range;

PointerInt n2=(n1 & 32) << \_\_3264divide; // check the 6th bit (or 7th in 64-bit)

// shift this down to the first bit

// Get the excess bits of our total range (will be 4 if n1 == 35) or zero if n2 is zero

PointerInt n5=theBit-((n1-\_\_3264minus1)\*n2); // move theBit along so that we don't over run the uint

// We now need from theBit bit to theBit+range mask - done fast because we have look up tables

PointerInt mask=(m\_TheBitMaskArray[n5]^m\_TheBitMaskArray[n5+range]);

PointerInt yextent=y+range;

if (yextent>m\_maxy) yextent=m\_maxy;

for (PointerInt yy=y; yy<yextent; yy++) { // Do all this for each y

//res=res|(m\_TheMap[index]&mask); // Can also test for non-zero here, but in a sparse matrix this slows things down too much

if (m\_TheMap[index]&mask) return true; // Can also test for non-zero here, but in a sparse matrix this slows things down too much

index+=m\_maxx;

}

// if (res) return true; else return false;

return false;

}

|  |  |  |  |
| --- | --- | --- | --- |
| int PositionMap::GetMapValue | ( | unsigned | *x*, |
|  |  | unsigned | *y* |
|  | ) |  |  |

References \_\_3264divide, \_\_3264minus1, m\_maxx, and m\_TheMap.

Referenced by GetTotalN(), and GetTotalSpace().

{

PointerInt theBit= x & \_\_3264minus1;

// divide by 32

// Calculate index:

PointerInt index= (x>> \_\_3264divide )+(m\_maxx\*y);

PointerInt res =((m\_TheMap[index] >> theBit) & 0x01); // 0-1

return int(res);

}

|  |  |  |  |
| --- | --- | --- | --- |
| int PositionMap::GetTotalN | ( | int | *x*, |
|  |  | int | *y* |
|  | ) |  |  |

References GetMapValue().

{

// This is a debug function which returns the total filled area of the map

int space=0;

for (int i=0; i<x; i++)

{

for (int j=0; j<y; j++)

{

if (GetMapValue(i,j)!=0) space++;

}

}

return space;

}

|  |  |  |  |
| --- | --- | --- | --- |
| int PositionMap::GetTotalSpace | ( | int | *x*, |
|  |  | int | *y* |
|  | ) |  |  |

References GetMapValue().

{

// This is a debug function which returns the total empty area of the map

int space=0;

for (int i=0; i<x; i++)

{

for (int j=0; j<y; j++)

{

if (GetMapValue(i,j)==0) space++;

}

}

return space;

}

|  |  |  |  |  |
| --- | --- | --- | --- | --- |
| void PositionMap::Init | ( |  | ) |  |

protected

References \_\_3264minus0, ClearMapValue(), m\_maxy, m\_TheBitMaskArray, m\_TheBitMaskArray2, and m\_xmaxx.

Referenced by PositionMap().

{

for (unsigned y=0; y<m\_maxy; y++)

{

for (unsigned x=0; x<m\_xmaxx; x++)

{

ClearMapValue(x,y);

}

}

// I guess there is a neater way to do this, but this works at least!

m\_TheBitMaskArray[0]=0;

for (int i=1; i<\_\_3264minus0; i++) {

m\_TheBitMaskArray[i]=m\_TheBitMaskArray[i-1]+(PointerInt(1) << (i-1));

}

for (int i=0; i<\_\_3264minus0; i++) {

m\_TheBitMaskArray[i]^= 0xFFFFFFFFFFFFFFFF;

m\_TheBitMaskArray2[i]= ~m\_TheBitMaskArray[i]; // 1s compliment

}

#ifdef \_\_64BIT

m\_TheBitMaskArray[\_\_3264minus0] = 0xFFFFFFFFFFFFFFFF;

#else

m\_TheBitMaskArray[\_\_3264minus0] = 0xFFFFFFFF;

#endif

m\_TheBitMaskArray2[\_\_3264minus0]= 0;

}

|  |  |  |  |
| --- | --- | --- | --- |
| void PositionMap::SetMapValue | ( | unsigned | *x*, |
|  |  | unsigned | *y* |
|  | ) |  |  |

References \_\_3264divide, \_\_3264minus1, m\_maxx, and m\_TheMap.

{

PointerInt theBit= x & \_\_3264minus1;

// divide by 32

// Calculate index:

PointerInt index= (x>> \_\_3264divide )+(m\_maxx\*y);

m\_TheMap[index] = m\_TheMap[index] | (PointerInt(0x01) << theBit);

}

---

## Member Data Documentation

Landscape\* PositionMap::m\_ALandscape

protected

Referenced by PositionMap().

PointerInt PositionMap::m\_maxx

Referenced by ClearMapValue(), GetMapDensity(), GetMapDensity32(), GetMapDensity5x5(), GetMapPositive(), GetMapPositiveB(), GetMapValue(), PositionMap(), and SetMapValue().

PointerInt PositionMap::m\_maxy

Referenced by GetMapDensity(), GetMapPositive(), GetMapPositiveB(), Init(), and PositionMap().

PointerInt PositionMap::m\_TheBitMaskArray[65]

Referenced by GetMapDensity(), GetMapPositive(), GetMapPositiveB(), and Init().

PointerInt PositionMap::m\_TheBitMaskArray2[65]

Referenced by GetMapDensity(), GetMapPositive(), and Init().

PointerInt\* PositionMap::m\_TheMap

Referenced by ClearMapValue(), GetMapDensity(), GetMapDensity32(), GetMapDensity5x5(), GetMapPositive(), GetMapPositiveB(), GetMapValue(), PositionMap(), SetMapValue(), and ~PositionMap().

PointerInt PositionMap::m\_xmaxx

Referenced by Init(), and PositionMap().

---

The documentation for this class was generated from the following files:

- positionmap.h
- PositionMap.cpp


- PositionMap
- Generated on Thu Jan 10 2013 13:15:37 for ALMaSS Skylark ODdox by
   1.8.1.1
